# 2309. Impact of Stopping Asymptomatic SARS-Co-V-2 Admission Testing: An Autoregressive Integrated Moving Average (ARIMA) Model Analysis at Stanford Healthcare, 2023

**DOI:** 10.1093/ofid/ofad500.1931

**Published:** 2023-11-27

**Authors:** Guillermo Rodriguez-Nava, Lucy S Tompkins, Karen McIntyre, Bryan Bohman, Jorge Salinas

**Affiliations:** Stanford University School of Medicine, Palo Alto, California; Stanford University, PORTOLA VALLEY, California; Stanford Healthcare, Stanford, California; Stanford School of Medicine, Emerald Hills, California; Stanford University, PORTOLA VALLEY, California

## Abstract

**Background:**

Admission SARS-Co-V-2 testing for asymptomatic patients has been implemented in most US hospitals. However, testing patients with a low pretest probability may lead to more false positives than true positives, inappropriate isolation and delays in care. We recently deimplemented asymptomatic admission testing at Stanford Healthcare. Here, we model the impact of this policy change on the number of false-positive COVID-19 cases.

**Methods:**

We retrieved data for inpatients with a positive SARS-Co-V-2 test at Stanford Healthcare from January 2020–February 2023. The Augmented Dickey-Fuller (ADF) test was used to determine if the time series was stationary. Autocorrelation function (ACF) and partial autocorrelation function (PACF) plots were generated to determine potential parameters for the ARIMA model. An automated algorithm was used to determine the optimal parameters for the final ARIMA model, which included 'step' and 'ramp' exogenous variables to account for policy interventions. We calculated the number of false positives by using infectiousness data previously collected using a SARS-Co-V-2 strand specific assay.

**Results:**

The number of inpatient COVID-19 cases at Stanford Healthcare during January 2020–February 2023 is illustrated in **Figure 1**. The ADF statistic was -4.48 (p< .001), indicating that the time series was stationary (data transformation not required). **Figure 2** shows the ACF and PACF plots. **Figure 3** shows the values predicted by our final ARIMA model in absence of the intervention (counterfactual) compared to observed values. Stopping asymptomatic SARS-Co-V2 admission testing was associated with a decrease of 1,396 and 518 SARS-CoV-2 positive cases in January and February 2023. Using a 9% active SARS-Co-V-2 infection in asymptomatic patients at our institution; we avoided 1,271 and 471 false positive cases in January and February 2023, although 125 and 47true positive cases may have been missed. Healthcare-associated COVID-19 cases did not change.

**Figure 1.**
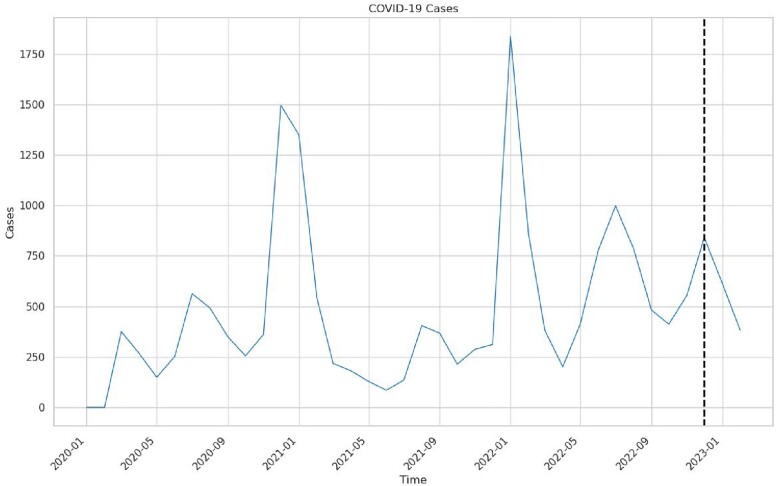
COVID-19 cases at Stanford Healthcare during January 2020–February 2023

**Figure 2.**
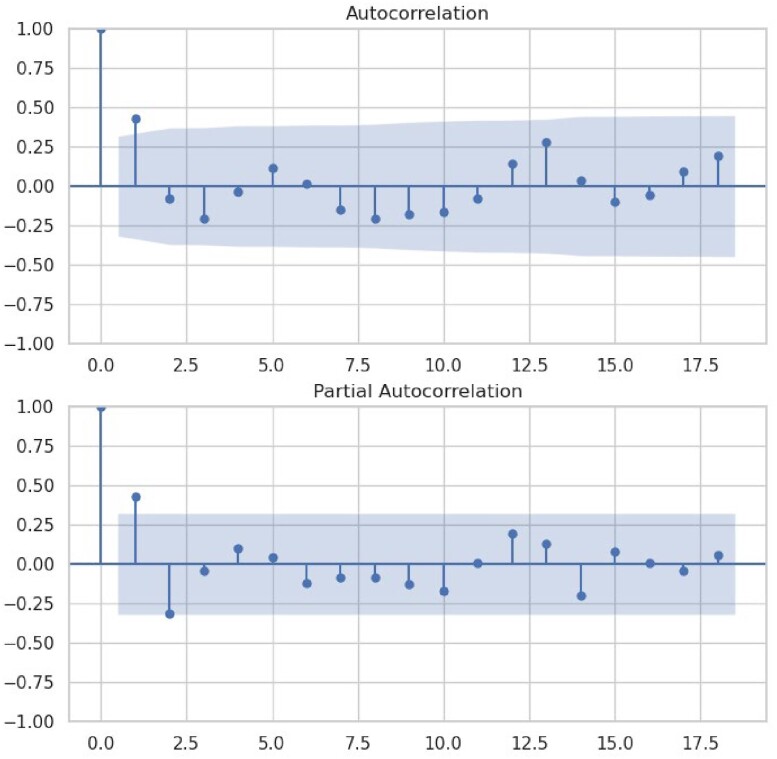
Autocorrelation function (ACF) and partial autocorrelation function (PACF) plots.

Figure 2 Caption We first used auto_arima to fit the ARIMA model with order (0, 0, 1). However, we noticed a seasonal trend with higher cases in September and January, so we included seasonal_order (1, 1, 1, 12) parameters in the final model.

**Figure 3.**
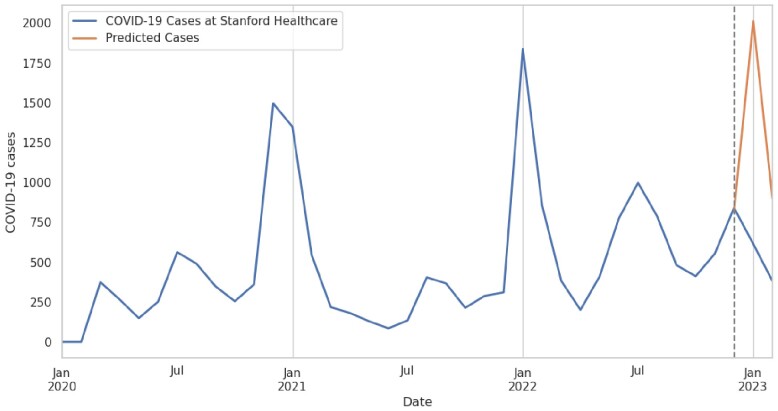
Final ARIMA model plot

The model had an intercept of 477.7352 and a ma.L1 coefficient of 0.4978, with a statistically significant sigma2 value of 1.204e+05. The Ljung-Box (L1) had a high probability of 0.75, indicating no evidence of autocorrelation, and the Jarque-Bera (JB) had a low probability of 0.00, indicating non-normality of the residuals. Heteroskedasticity was observed with a p-value of 0.07, suggesting the presence of non-constant variance in the error terms.

**Conclusion:**

The policy change to stop asymptomatic SARS-Co-V-2 admission testing was associated with an immediate decrease of false-positive COVID-19 cases, conserving healthcare resources and avoiding unnecessary isolation without compromising patient safety.

**Disclosures:**

**All Authors**: No reported disclosures

